# Climate change and plant dispersal along corridors in fragmented landscapes of Mesoamerica

**DOI:** 10.1002/ece3.672

**Published:** 2013-07-30

**Authors:** Pablo A Imbach, Bruno Locatelli, Luis G Molina, Philippe Ciais, Paul W Leadley

**Affiliations:** 1 Climate Change Program, CATIECosta Rica; 2 CIRAD UPR Forest Ecosystem ServicesMontpellier, France; 3 CIFOR ENV ProgramBogor, Indonesia; 4 IPSL – LSCE, CEA CNRS UVSQ, Centre d'Etudes Orme des Merisiers91191, Gif sur Yvette, France; 5 Univ Paris-Sud, ESE Laboratory, UMR 8079, CNRS / Univ Paris-Sud / AgroParisTech91405, Orsay Cedex

**Keywords:** Biodiversity conservation policy, biological corridors, cellular automaton, climate change adaptation, connectivity, Holdridge, landscape fragmentation, MAPSS, migration, species dispersal

## Abstract

Climate change is a threat to biodiversity, and adaptation measures should be considered in biodiversity conservation planning. Protected areas (PA) are expected to be impacted by climate change and improving their connectivity with biological corridors (BC) has been proposed as a potential adaptation measure, although assessing its effectiveness remains a challenge. In Mesoamerica, efforts to preserve the biodiversity have led to the creation of a regional network of PA and, more recently, BC. This study evaluates the role of BC for facilitating plant dispersal between PA under climate change in Mesoamerica. A spatially explicit dynamic model (cellular automaton) was developed to simulate species dispersal under different climate and conservation policy scenarios. Plant functional types (PFT) were defined based on a range of dispersal rates and vegetation types to represent the diversity of species in the region. The impacts of climate change on PA and the role of BC for dispersal were assessed spatially. Results show that most impacted PA are those with low altitudinal range in hot, dry, or high latitude areas. PA with low altitudinal range in high cool areas benefit the most from corridors. The most important corridors cover larger areas and have high altitude gradients. Only the fastest PFT can keep up with the expected change in climate and benefit from corridors for dispersal. We conclude that the spatial assessment of the vulnerability of PA and the role of corridors in facilitating dispersal can help conservation planning under a changing climate.

## Introduction

Climate change is a major threat to biodiversity (MEA 2005; Pereira et al. 2010, 2012). It will affect the geographic ranges of species (Walther et al. 2002; Parmesan and Yohe 2003) and thus, ecosystems species, populations, and communities. Future distribution of species and ecosystems depends on the ability of plants to migrate (Pitelka et al. 1997). Plant migration is an essential response of vegetation to climate change, as the capacity for in situ responses (persistence and genetic adaptation) (Thuiller et al. 2008) may be exceeded by the rate of climate change (Midgley et al. 2007). Many studies on climate change and ecosystems consider unlimited dispersal or no dispersal (Thomas et al. 2004; Jetz et al. 2007; Araújo et al. 2011), in particular global studies, because of their coarse resolution (Kirilenko et al. 2000).

Habitat fragmentation may reduce plant migration capacity by reducing suitable habitat for successful colonization (Pitelka et al. 1997; Higgins et al. 2003b). Biological corridors (BC) can facilitate migration between valuable biodiversity areas (e.g., protected areas [PA]). Corridors facilitate movement between habitat patches, especially the movement of invertebrates, nonavian vertebrates, and plants (Gilbert-Norton et al. 2010; Hannah 2011).

The spatial configuration of BC determines their efficiency in facilitating dispersal and migration between PA, depending on the location of these areas and current and future climate patterns. For example, in a region where climate change will shift species distribution toward the poles, corridors oriented longitudinally may be ineffective. There is no simple rule for deciding how to design corridor networks for climate change adaptation (Phillips et al. 2008; Vos et al. 2008; Hole et al. 2011). Altitudinal corridors have been recommended (Innes et al. 2009), as well as latitudinal corridors, for example, in boreal areas (Roberts et al. 2009).

Biodiversity is highly threatened in the Mesoamerican region, mainly by anthropogenic activities (DeClerck et al. 2010). The Mesoamerican Biological Corridor (MBC) is a regional network of PA and corridors that has been created as a multilateral response to help biodiversity conservation (CCAD-UNDP/GEF 2002).

As biodiversity conservation policies will increasingly have to address climate change (Brooke 2008; Sutherland et al. 2009), there is a need to incorporate forecasting models into decision making (Thuiller et al. 2008), for example, to understand how to prioritize corridors for adaptation to climate change (Heller and Zavaleta 2009; Hannah 2011). Understanding the role of PA and corridors in facilitating plant migration requires fine-scale and spatially explicit modeling approaches that simulate migration across landscapes under climate change scenarios (Pearson 2006). There is also a need to take into account uncertainties by considering different climate change scenarios and different models of the temporal and spatial dynamics of species (i.e., species plasticity and dispersal capacity) (Bellard et al. 2012; Cheaib et al. 2012).

The objective of this study is to assess how corridors could facilitate plant dispersal between PA under climate change scenarios in Mesoamerica. For this, we developed a cellular automaton model for plant dispersal with a spatial resolution of 4.5 km (pixel size) in combination with different habitat models and species dispersal parameters.

Our approach focuses on species dispersal and combines habitat models to predict potential ranges of future suitable climates with a cellular automaton to model the dispersal process, as suggested by Thuiller et al. (2008). This focus on dispersal addresses the objective of understanding the role of corridors in allowing dispersal at the regional scale rather than studying vegetation dynamics under climate change.

Similar spatially explicit modeling of impacts of climate change on plant species based on cellular automaton have been applied for studying the dispersal of one plant species in a changing climate in the United Kingdom at 10 km resolution (Carey 1996), for calculating the probability of migration of four tree species in the United States at 1 km resolution (Schwartz et al. 2001) and to identify multiple corridors for facilitating the dispersal of Proteaceae in South Africa at 1.7 km resolution (Williams et al. 2005). Treeline shifts (Dullinger et al. 2004) and plant dispersal effects on range distributions (Engler et al. 2009) under climate change have also been studied over smaller areas (54 and 700 km^2^, respectively) and with higher spatial resolution (30 and 25 m pixel size, respectively).

## Methods

### Study area

Mesoamerica comprises the continental land within 6.5–22°N and 76.5–99°W, covering one million square kilometers between southern Mexico and Panama (running across Guatemala, El Salvador, Honduras, Nicaragua, and Costa Rica). A mountain range running from South to North close to the Pacific coast is its main topographical feature and shapes its regional climate pattern with higher precipitation on the eastern slopes (Hastenrath 1967).

Its highly variable precipitation contrasts with an annual temperature cycle with small amplitude when compared to temperate areas. The seasonal pattern in precipitation is driven by the ITCZ (Inter-Tropical Convergence Zone) with easterly winds increasing seasonality when hitting the mountain ranges (Nieuwolt 1977), resulting in a bimodal patter with maxima in June and September–October (Magaña et al. 1999) and high interannual variability (Aguilar et al. 2005).

Mesoamerica is a biodiversity hotspot (Myers et al. 2000) with high plant and mammal species richness (Greenheck 2002) and an important role for biogeography, as it served as a species bridge between South and North during the Great American Biotic Interchange (Stehli and Webb 1985). There are over 12,000 known species of amphibians, birds, mammals, and reptiles (DeClerck et al. 2010) and over 5000 endemic species of vascular plants (Greenheck 2002). It has four biomes and 19 ecoregions with different natural and anthropogenic disturbance regimes.

**Figure 1:**
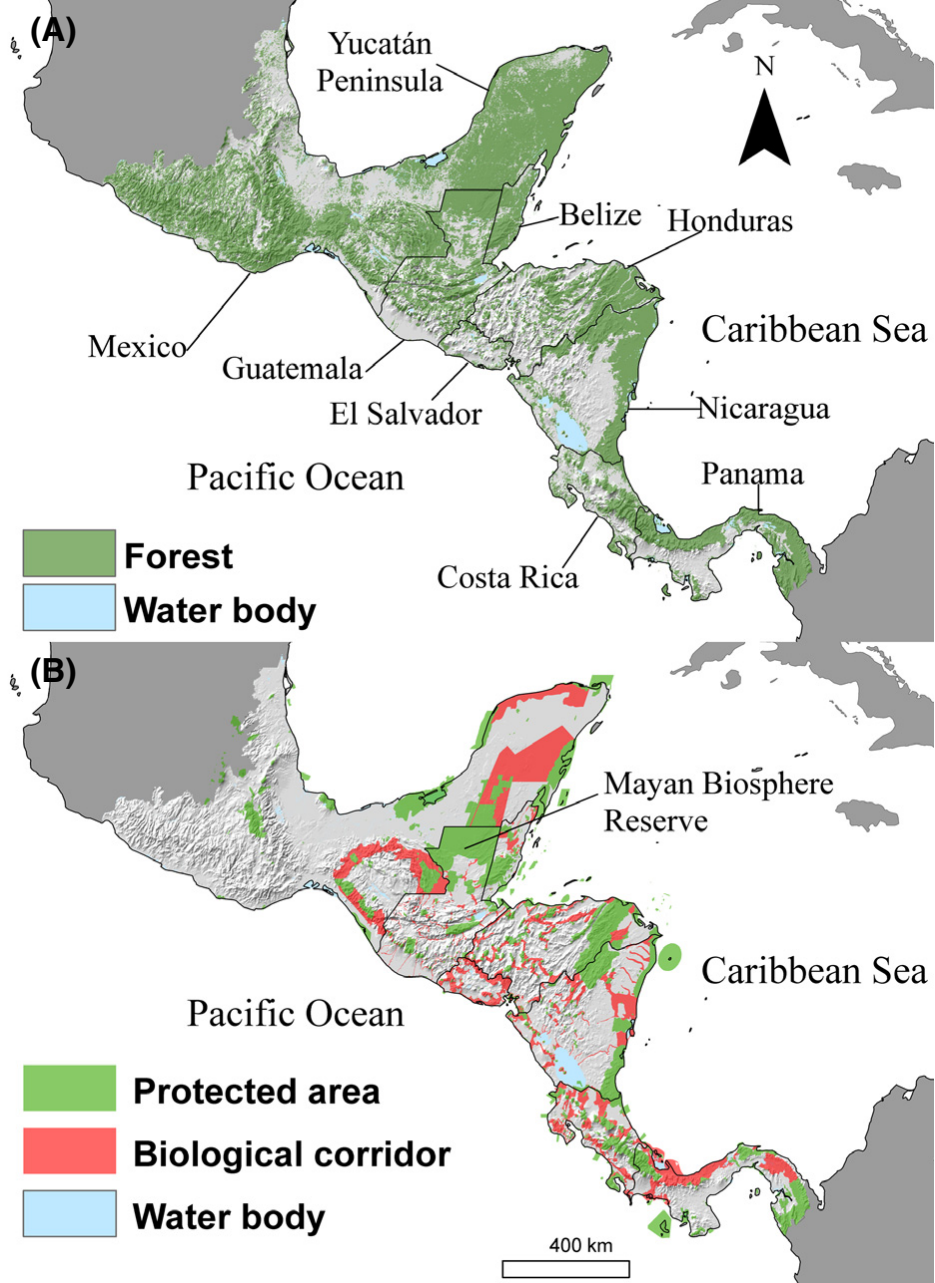
Land cover (2003) (A) and the Mesoamerican Biological Corridor (MBC) (B) (CCAD 2001; CCAD and WB 2003) (Mollweide projection).

Regional integration of conservation policies was boosted in 1992 with the creation of the Central American System of Protected Areas (SICAP) and later on, in 1997, of the MBC. The MBC aims at improving the connectivity of PA at regional scale while improving human livelihoods. Despite conservation efforts, isolation of PA can put the system's network functions at risk (Sánchez-Azofeifa et al. 2003) which are threatened by land-use change as 43% of the region is under productive land uses (i.e., agriculture, pastures, or urban), with pastures tripling agricultural areas (DeClerck et al. 2010) (Fig. 1). Furthermore, some biomes are poorly represented in conservation areas (i.e., 3% of the tropical dry broadleaf forests and there is no protection for xeric shrublands) (DeClerck et al. 2010).

Mesoamerica is expected to be a climate change hotspot among tropical areas (Giorgi 2006). A consistent drying signal across the region is a characteristic feature of future global scenarios (Neelin et al. 2006; Sheffield and Wood 2008) even at the seasonal scale (Rauscher et al. 2008). Between 1961 and 2003, temperatures extremes (maximum and minimum) have increased as has the amount of precipitation falling during extreme events (Aguilar et al. 2005). Observed precipitation trends vary depending on location (Malhi and Wright 2004), but total annual regional rainfall has remained constant (Aguilar et al. 2005).

### Analysis of climate change velocity

We used four climate scenarios combining two contrasted emission scenarios (A2A and B2A, higher and lower greenhouse gas emissions respectively) and two climate models [HADCM3 and CCCMA, from Hadley Centre (U.K.) and the Canadian Centre for Climate Modelling and Analysis, respectively]. Temperature and precipitation from climate scenarios downscaled at 2.5-arc-minute resolution were taken from the WorldClim database (Hijmans et al. 2005). On average over the region, climate scenario HADCM3 had the highest temperature increase of 2.2–3.2°C in 2050 (depending on the emission scenario), whereas CCCMA showed a maximum increase of 2.0–2.1°C. Future scenarios of precipitation, with CCCMA (in 2050), showed increased annual precipitation between 445 and 539 mm on the Pacific watershed and southern countries (Costa Rica and Panama) and a reduction of 399–679 mm on the Caribbean watershed depending on the emission scenario (HADCM3-B2A showed similar changes). HADCM3-A2A showed a reduction in precipitation across the whole region (up to 1948 mm on the Caribbean watershed).

We first estimated climate change velocity (Loarie et al. 2009) to explore the four climate scenarios used for analysis. Climate change velocity estimates for each pixel the change in a climate variable per unit of time related to its surrounding gradient in climate, resulting in the speed (km/year) that one should move in the future to find the same climate as in the present. It is estimated as the ratio between spatial gradients (of temperature or precipitation) and temporal gradients of future climate (on average over the 1990–2050 period). The spatial gradient is the maximum gradient in the baseline climate over a 3 × 3 grid cell window. The temporal gradient is the change per year of the climate variable. We calculated two velocities of change (for annual mean temperature and precipitation) and mapped the maximum of the two velocities as a proxy for the constant dispersal rate needed to find a reference climate analog under future climate conditions.

### Model: Overview

We developed a cellular automaton (a spatially explicit dynamic model) over Mesoamerica using a spatial resolution of 2.5 arc minutes (∼4.5 km pixel with a total of 51,000 pixels) and a time step of 10 years from 1990 to 2050.

Our simulation on how corridors can facilitate species dispersal is based on potential vegetation types determined by climate (Holdridge 1947; Neilson 1995) and on the modification of suitable environmental ranges for a given vegetation type by climate change. We assumed that when climate changes and becomes unsuitable for a vegetation type, its species can disperse isotropically to nearby locations depending on their capacity and landscape fragmentation. Only pixels that are under natural vegetation are suitable for plant dispersal and dispersal is assumed to be the only response of plants to a changing climate. We ignored other responses such as persistence or adaptation over generations (Midgley et al. 2007) as well as species differences in the leading and trailing edges of its distribution range (Hampe and Petit 2005). In the migration process, we focused on dispersal and ignored recruitment, establishment, mortality, or changes in abiotic interactions between species (Guisan and Thuiller 2005).

### Model: Vegetation types

We assumed that all vegetation types and their species are in equilibrium with our reference climatology, therefore, ignoring ongoing range shifts due to previous climate change or other drivers (Araújo and Pearson 2005). Two vegetation models were used for predicting vegetation types in equilibrium with climate: MAPSS (Mapped Atmosphere Plant Soil System), a mechanistic model (Neilson 1995), and Holdridge, a correlative model (Holdridge 1947). Both selected models determine vegetation types using climate variables (MAPSS also uses soil data) and allow for assessments under future climate conditions. MAPSS simulates vegetation in equilibrium with a site energy and water constraints and estimates the leaf area index (LAI) of each life form (grass, shrub, or tree) that can be supported given available soil water. A set of rules based on LAI, phenology, leaf form, and thermal zones defines the type of vegetation (Neilson 1995). The Holdridge life zones are empirically based “conditions for ecosystems functioning” (Lugo et al. 1999) based on the combination of classes for precipitation, biotemperature, potential evapotranspiration, and elevation. Both models have been used for Mesoamerica (Holdridge 1967; Imbach et al. 2010) and their performance compared for simulations under climate change scenarios (Yates et al. 2000).

### Model: Plant dispersal

According to paleobotanical studies based on fossil pollen data, rapid spread (on the order of 100–1000 m/year) was typical of tree species in postglacial warming in Europe and North America (Clark et al. 1998; Malcolm et al. 2002; Pearson 2006). As this migration is too rapid to be explained by diffusion process (Clark et al. 1998), two main explanations have been given to this paradox (the so called Reid's Paradox): the existence of small refuges having facilitated recolonization and the existence of rare long-distance dispersal (LDD). LDD results from a few diasporas moving long distances, in association with diffusion, in which most diasporas move short distances (Ronce 2001; Nathan 2006; Midgley et al. 2007).

A simple approach for including dispersal into species distribution modeling is to use an estimate of distance per unit of time for each plant species (Guisan and Thuiller 2005). This approach has been used in several spatial models of plant migration (Kirilenko and Solomon 1998; Williams et al. 2005), generally with a stochastic approach when rare LDD is considered (Dyer 1995). For example, Morin and Thuiller (2009) used dispersal rates for species taking into account LDD events and the probability of successful establishment.

Previous modeling studies considered plant dispersal through contiguous pixels of suitable habitat (Kirilenko and Solomon 1998; Morin and Thuiller 2009), through noncontiguous pixels (Carey 1996; Schwartz et al. 2001), or both: diffusion through contiguous pixels and LDD through noncontiguous pixels (Dyer 1995; Williams et al. 2005). In the case of contiguous pixel dispersal, plants can only reach suitable pixels that are connected to their origin pixel through other suitable pixels. It can be the case of plants whose seeds are transported by forest mammals avoiding nonforested areas. In the noncontiguous case, plants can move to any suitable pixels within their reach as can be the case of seeds transported by birds or wind over rather long distances. Even though landscape structure and the presence of corridors influence less LDD than diffusion (Pearson and Dawson 2005); corridors can act as stepping stones for LDD across consecutive time steps. For this reason, we considered both diffusion through contiguous pixels and LDD through noncontiguous pixels. When implemented in the model at a particular spatial resolution (4.5 km pixel in this case) we are also assuming a threshold below which forest patches cannot function as stepping stones due to their size (forest patches smaller than the selected resolution are not accounted for) or the distance between forest patches (forest patches separated by distances below the nondiagonal and diagonal pixel size are not accounted for). Depending on the species this could lead to conservative estimates.

### Model: Dispersal rules

We developed a knowledge-based model for simulating dispersal, composed by a set of propositions and an inference engine using fuzzy logic. Fuzzy logic is increasingly used in disciplines such as environmental modeling because it can handle imprecise or incomplete knowledge (Phillis and Andriantiatsaholiniaina 2001; Ervin 2006; Lawry 2006). Fuzzy models can apply expert knowledge (e.g., heuristic rules) to ecological data for inferring solutions and solving complex problems (Mackinson 2001; Shepard 2005). The knowledge at the core of the model can be described in natural language and fuzzy set theory can handle the uncertainties associated with this natural language (Eierdanz et al. 2008). Thus, even though the inference model and its outputs are numerical, the core of the model is qualitative and its empirical structure can be explained easily to policymakers or other stakeholders (Mackinson 2001; Reynolds et al. 2003). At the heart of the fuzzy set theory is the notion of possibility, which defines the degree of truth of a statement, or how possibly an event may occur, rather than its probability of occurrence (Cox 1994). Possibility is measured with the degree of membership in a set, for example, the set of successful dispersal events between two pixels. Possibility is measured by a continuous value between 0 and 1 (0 meaning completely impossible, 1 meaning completely possible, and other values meaning intermediate degrees of possibility).

**Figure 2:**
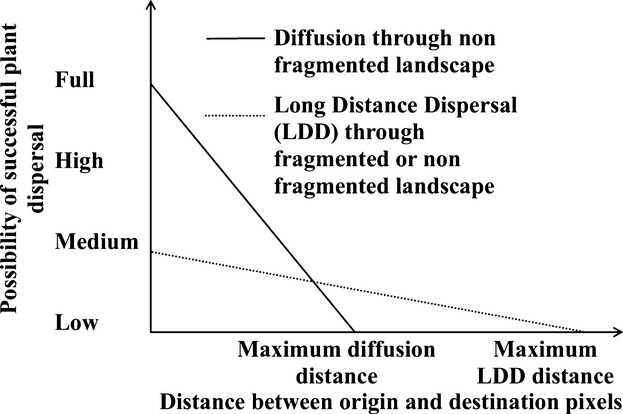
Possibility for a plant to migrate between two pixels, depending on euclidean distance between pixels and migration mode (LDD or diffusion).

We used the following rules for the dispersal model: (1) the possibility of diffusion from one pixel to another through nonfragmented landscape decreases with the distance between pixels, it is high at small distance and is null when distance reaches a maximum diffusion distance, (2) the possibility of LDD from one pixel to another through a fragmented or nonfragmented landscape is lower than the possibility of simple diffusion; it decreases with increasing distance between pixels, it is low at short distance and is null when distance reaches a maximum LDD distance (maximum dispersal distance is higher for LDD than for diffusion) (Fig. 2). Existing dispersal kernels in the literature are usually nonlinear functions of distance from the source area (Higgins and Richardson 1999; Higgins et al. 2003a,b,c), which in our case could lead to a dispersal underestimation at both short and long distances. However, our 10-year time-step estimates a dispersal event at longer distances (at least 500 m) which can result from the combined action of several dispersal vectors and whose aggregated kernels could tend to linear (after 500 m) (Nathan et al. 2008). In this sense, species that have smaller dispersal distances or specific vector kernels are not well represented. The model presented is rather phenomenological and not a mechanistic approach to species-specific dispersal.

### Model: Plant functional types

Plant dispersal rates are highly variable, in part because of the diversity of dispersal modes (Vittoz and Engler 2007). For example, previous modeling studies have used migration rates of 1–10 km/year for tropical plants (Kirilenko and Solomon 1998), 1–10 km/year for North American boreal and temperate trees (Morin and Thuiller 2009), 200 m/year for a tree species (Dyer 1995), 170 m/year for ant- and rodent-dispersed species, and 400 m/year for wind-dispersed species (Williams et al. 2005).

We aggregated plant species into a few plant functional types (PFTs), similar to the grouping made in dynamic global vegetation models (Gerten et al. 2004; Krinner et al. 2005), except that the functional types considered in most of these models are phenological and physiological, but do not incorporate dispersal and migration traits (Neilson et al. 2005; Thuiller et al. 2008). For our model, we assumed that a PFT is defined by its climatic suitability (i.e., vegetation type defined by MAPSS or Holdridge types every 10 years) and its dispersal capacity (by diffusion and LDD). Because of the lack of data on plant dispersal rates in Mesoamerica, we considered that seven PFTs (each one with a different diffusion and LDD rates, Table 1) exist for each vegetation type and are assumed to be in equal proportion under current climate conditions (therefore assuming equal number of species per dispersal rate category for each vegetation type). The assumption is that any real species (we did not account for any specific one here) would be represented by each PFT (which are defined by its dispersal rate and climate suitability).

**Table 1:** Diffusion and long-distance dispersal (LDD) rates (km/year) of hypothetical species in each plant functional type (PFT)

PFT	Diffusion	LDD
A	0.05	0.1
B	0.12	0.25
C	0.25	0.5
D	0.25	1
E	1.25	2.5
F	2.5	5
G	5	10

Each PFT was modeled independently and assumed to be restricted to its vegetation type range (defined by MAPSS and Holdridge models under current and future climate). A PFT from a specific vegetation type will move from its original location to any pixel with similar vegetation type at each time step and its presence recorded if the pixel is within the PFT dispersal range (see section on simulation and analysis for details on the estimation of presence values). We did not use rates whose dispersal distance is lower than the size of a pixel for the simulation period, but for the slowest PFTs (PFTs A and B for diffusion and A for LDD), a dispersal event can only occur after two time steps (time required to cover the distance to a nearby pixel at their dispersal speed).

### Model: Simulation

We simulated the presence of PFTs in each pixel with a fuzzy variable *μ* (*x*, *p*, *t*) (continuous between 0 and 1) representing the possibility that a PFT *p* is present in a pixel *x* in a time step *t* (10-year time steps). At the beginning of the simulation, we assumed that all plant types suited to the initial climate are present in pixels under natural vegetation, therefore:

*μ*(*x*, *p*, 1) = 1, if *p* is suited to the climate of *x* during time step 1; *μ*(*x*, *p*, 1) = 0 otherwise. These binary values correspond to the beginning of the simulation, but during the simulation, the possibility of a PFT being present in a pixel can take any value between 0 and 1.

We used two logical rules for the fuzzy model:

- Rule 1: “A plant is present in a pixel during a given time step if it was present during the previous time step or if it has been able to disperse from another pixel during this time step”. In fuzzy terms, it means that:

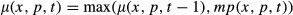


where *mp*(*x*, *p*, *t*) is the possibility of *p* dispersing to *x* during time step *t*. The *max* operator corresponds to the *or* logical operator.

(ii) Rule 2: “Migration of *p* to *x* occurs if at least one pixel of the landscape hosts the plant type *p* and *p* can disperse from this pixel to *x*.” The landscape is delimited by the maximum dispersal distance for each plant type at each time step. In fuzzy terms, it means that:

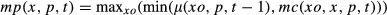


where *xo* is a pixel in the landscape and *mc* (*xo*, *x*, *p*, *t*) is the possibility of dispersal of plant type *p* from *xo* to *x*, depending on dispersal rates and landscape fragmentation, as defined in Figure 2. The *min* operator corresponds to the *and* logical operator in rule 2. The *max* operator is applied to all possible origin pixels *xo* in the landscape.

It is important to note that the possibility of dispersal accounts for dispersal rates every 10 years (the simulation time step) and therefore assumes how both dispersal probability functions and frequency of dispersal events (i.e., per year) aggregate at the model time step. For example, the model considers that the maximum diffusion distance of the slowest PFT is 0.5 km over 10 years and the maximum LDD distance in 1 km over 10 years (Table 1). Slower species require aggregating dispersal rates over two time steps (20 years) in order for the species to leave one pixel and land in a contiguous one. A similar approach is used for LDD in order for a species to reach a noncontiguous pixel over more than one time step. The choice of a 10-year time step is explained by limits in computing time at shorter time steps. We recognize that, in fragmented landscapes, a shorter time step would have produced different results for LDD, as a 10-year time step allows dispersal (between suitable pixels and over areas of nonsuitable pixels) over longer distance than a 1-year time step.

The impacts of climate change on PA were estimated as the mean change over the protected area in the presence of the seven functional types under different climates, policy scenarios, and vegetation models, using equation (1):


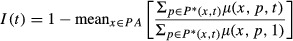
(1)

where *I*(*t*) is the impact at time step *t*, *PA* is the set of pixels in PA, and P*(*x*, *t*) is the set of PFTs suited to climate *x* at time step *t*. The formula estimates the mean decrease in PFT presence at the end of the analyzed period compared to the initial PFT presence. The full impact range at the last time step (in 2050) (from total PFT loss to no change in PFT presence) is divided into quintiles that define the five categories to be mapped (very low, low, medium, high, and very high).

During the simulations, we tracked dispersal pathways (e.g., succession of different dispersal events for the same PFT during the simulation period) for each PFT by recording the PFT, the origin and destination pixels, the decade, and the degree of possibility of this dispersal. The number of dispersal events per simulation ranged from 2 × 10^6^ to 5 × 10^6^ depending on the simulation. The number of different dispersal pathways was estimated around 2 × 10^8^ (10^8^ to 3.10^8^ was the 90% confidence interval estimated from a subset of origin pixels). The number of pathways is high because if a PFT disperses from one pixel to 10 pixels during each time step, there may be up to 100 different pathways after two time steps. Due to computational constraints, we did not record and analyze all pathways, but we used the information on dispersal events to reconstruct a subset of 2 × 10^5^ pathways. Each pathway was associated with a possibility, defined as the minimum possibility of all dispersal events in the pathway. We analyzed how many pathways connected two PA through a corridor and the possibility that a pixel in a corridor was used for a migration pathway between two PA. The result is presented in a map of important corridors for the migration between PA.

### Analysis of the impacts of climate change with and without corridors

We mapped the impacts of climate change with and without corridors to analyze whether corridors reduce significantly the impacts of climate change on PA. Two regional conservation policy scenarios were evaluated: (i) strong conservation policies: vegetation is restored or conserved in BC and PA to its natural (pristine) state (and therefore providing suitable habitat), whereas vegetation outside BC or PA is conserved in its current state (suitable if covered by natural vegetation and unsuitable otherwise); (ii) weak conservation policies: vegetation is restored or conserved in PA and fully degraded outside PA (to unsuitable habitat), including in BC. Spatial data on current land use, PA, and BC were taken from existing regional maps (CCAD-UNDP/GEF 2002; Vreugdenhil et al. 2002).

### Analysis of explanatory variables

The dependence of impacts on policies (two cases), PFTs (seven cases), and vegetation models (two cases) was tested by analysis of variances (ANOVA). Significance level for the analyses was set at *P* < 0.01. We explored correlations between the impacts of climate change on PA and several explanatory variables; area, altitude mean, and range within each PA, current mean, and future change in temperature and precipitation. We also explored correlations between the importance of corridors and the same explanatory variables.

The explanatory power of the variables was analyzed using the hierarchical partitioning protocol (Chevan and Sutherland 1991). The protocol explores all possible multiple regressions and determines the influence of a single variable by averaging its influence on the models in which it appears (Mac Nally 2000). The hierarchical organization is needed to account for simpler models that are nested into complex ones while mitigating potential multicollinearity problems. Variances are partitioned so the independent contribution of a variable can be estimated. The incremental goodness of fit (by adding the variable) in the models are averaged to measure independent effects. The explanatory power of a variable (IE) is measured by its proportion on total independent effects (the joint effect of a variable with the others was not analyzed). We used a public domain package to perform this analysis (Mac Nally and Walsh 2004; Walsh and Mac Nally 2009). Additionally, to determine the most important variables, for each variable we generated a distribution of IE based on independently randomized values (1000 simulations). Variables with an observed IE that is extreme to the generated distribution (*Z*-score >1.65 or a 95% confidence limit) were considered the most important.

## Results

### Climate change velocity

**Figure 3:**
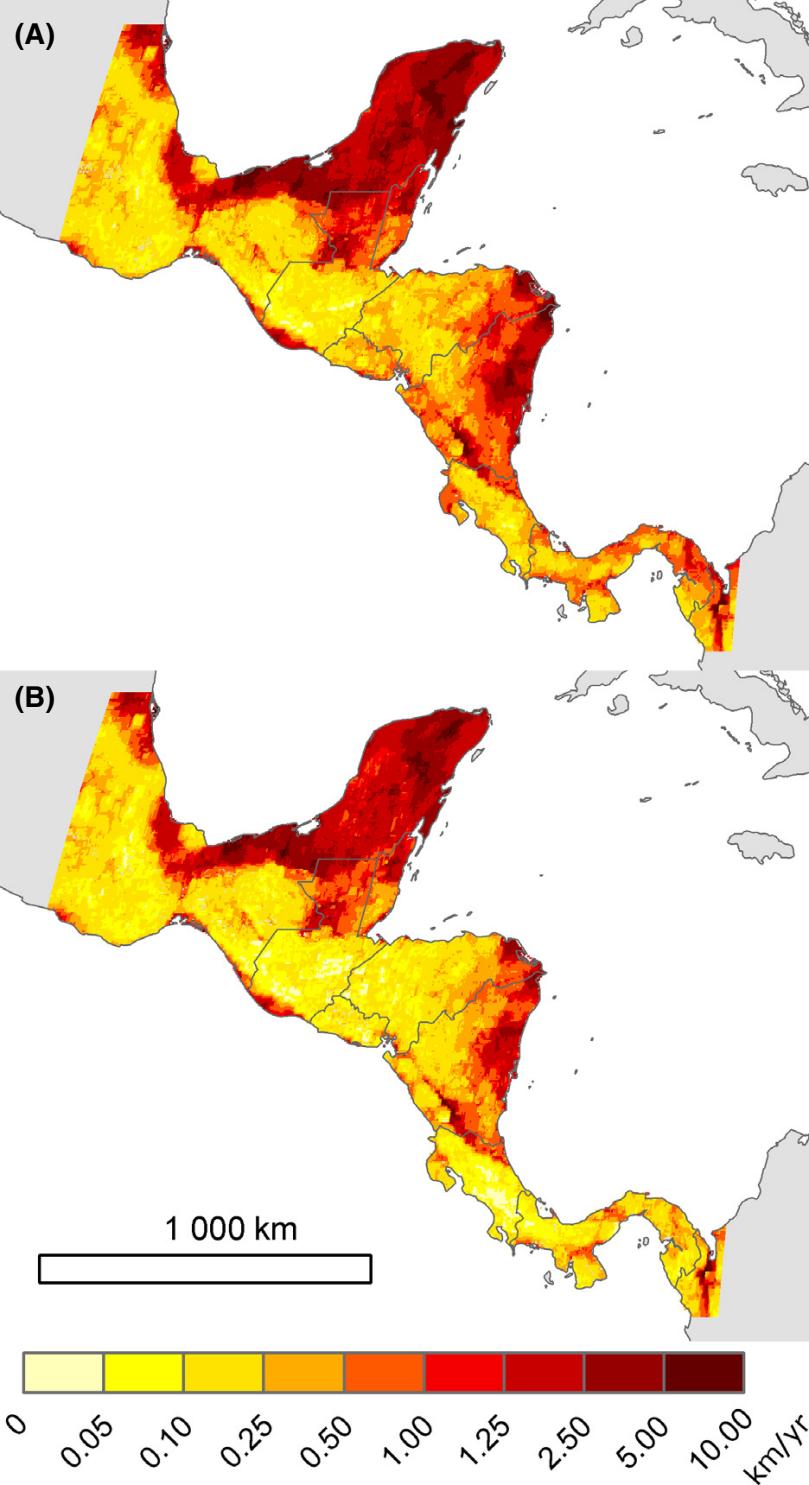
Velocity of climate change (km/year) for HADCM3-A2A (A) and CCCMA-B2B (B) scenarios (velocity classes according to dispersal rates for each plant functional type used in the study) (Mollweide projection).

High velocity of climate change was found in flat areas in the Atlantic coast of Nicaragua and the Yucatan peninsula in Mexico, with values up to 15 km/year under HADCM3-A2A in the 1990–2050 period (Fig. 3). Low velocity was observed in mountainous areas of Costa Rica, Honduras, Guatemala, and Mexico, where velocities were below 0.25 km/year for HADCM3-A2A and 0.1 for CCCMA-B2A. Correlation of temperature change velocity and slope had a range −0.44 to −0.45 across scenarios, whereas for precipitation velocity the correlation was lower (−0.3 to −0.34). In general, climate change velocity is higher for HADCM3 (max 15 km/year, mean 0.95 km/year) than CCCMA (max 10 km/year, mean 0.56 km/year) and higher for A2A (max 15 km/year, mean 0.86 km/year) than B2A (max 13 km/year, mean 0.67 km/year). On all scenarios, velocity of climate change is higher than 5 km/year in 1–2% of the pixels (lower threshold for the fastest PFT) and less than 0.1 km/year in 16–17% (upper threshold for the slowest PFT). Areas with velocities higher than the fastest PFT are insignificant. Figure 3 shows only scenarios with the maximum (HADCM3-A2A) and minimum (CCCMA-B2B) average velocity (a similar pattern is found in the other two scenarios). Under HADCM3-B2B and CCCMA-A2A/B2A scenarios, the change velocity is higher for precipitation than temperature in more than 90% of the area. For HADCM3-A2B, the velocity is higher for temperature than precipitation in 62% of the area.

### Modeled vegetation

**Figure 4:**
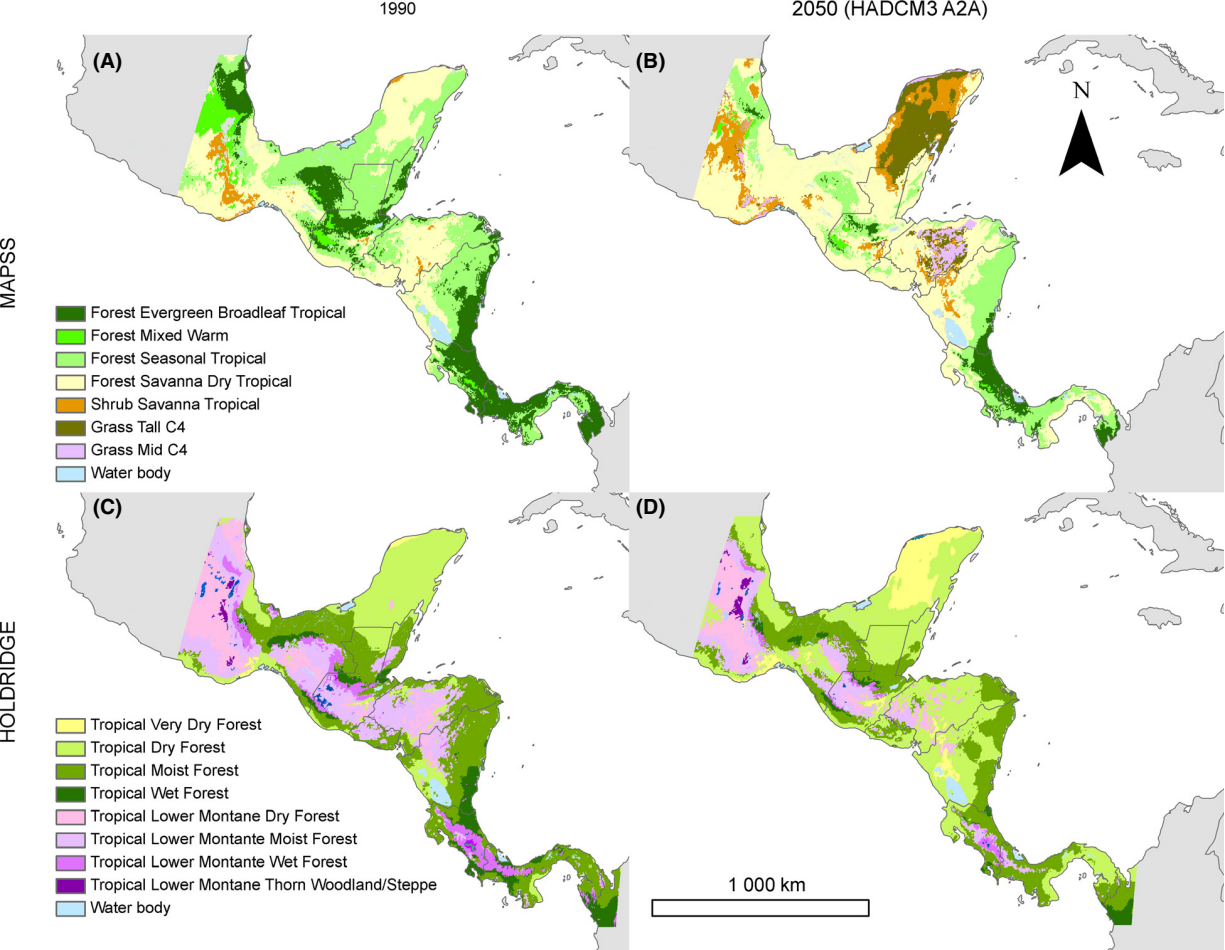
Distribution of potential vegetation types in 1990 and 2050 under the HADCM3 A2A climate change scenario, with MAPSS and Holdridge vegetation models (Mollweide projection). For simplicity the legend shows only major vegetation classes covering 98% of the area.

Current vegetation type classes differ on both vegetation models used. For example, in the Yucatan Peninsula, MAPSS distinguished between seasonal and dry forests, whereas Holdridge finds dry forests only, probably because Holdridge does not capture seasonal features (Yates et al. 2000). For the same reason, under future scenarios with MAPSS, forests in Yucatan and central Honduras are converted into grasses and shrubs (Fig. 4). Holdridge on the other hand differentiates more classes in mountain areas compared with MAPSS as it considers altitudinal belts for vegetation types.

### Impacts of climate change on PA without corridors

Impacts of climate change (*I*) on PA in the absence of corridors (“weak conservation” scenario) depend significantly (*P* < 0.001) on climate scenarios, vegetation models, and dispersal rates. Impacts are higher under HADCM3-A2A scenario, which is the one with the largest climate change (mean *I* = 0.55), than other scenarios (0.26 for both CCCMA scenarios and 0.32 for HADCM3-B2B), and higher with MAPSS model (0.46) than Holdridge (0.24). As expected, slow moving plant types are more impacted by climate change (e.g., 0.46 for type A) than fast-moving plant types (e.g., 0.20 for type G).

**Figure 5:**
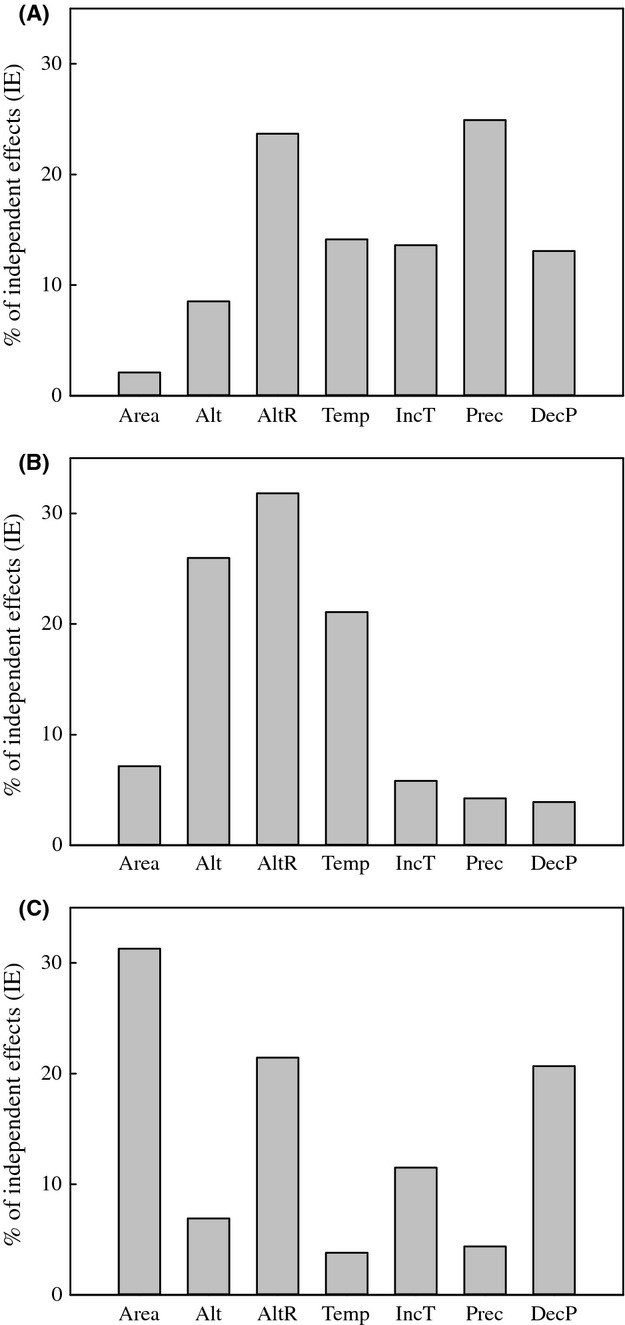
Percentage of independent effects for each variables explaining the impacts of climate change on protected areas (A), the benefit of protected areas from biological corridors (B), and importance of corridors (C). Alt, altitude; AltR, altitude range; Temp, temperature; IncT, increase in temperature; Prec, precipitation; DecP, decrease in precipitation.

**Figure 6:**
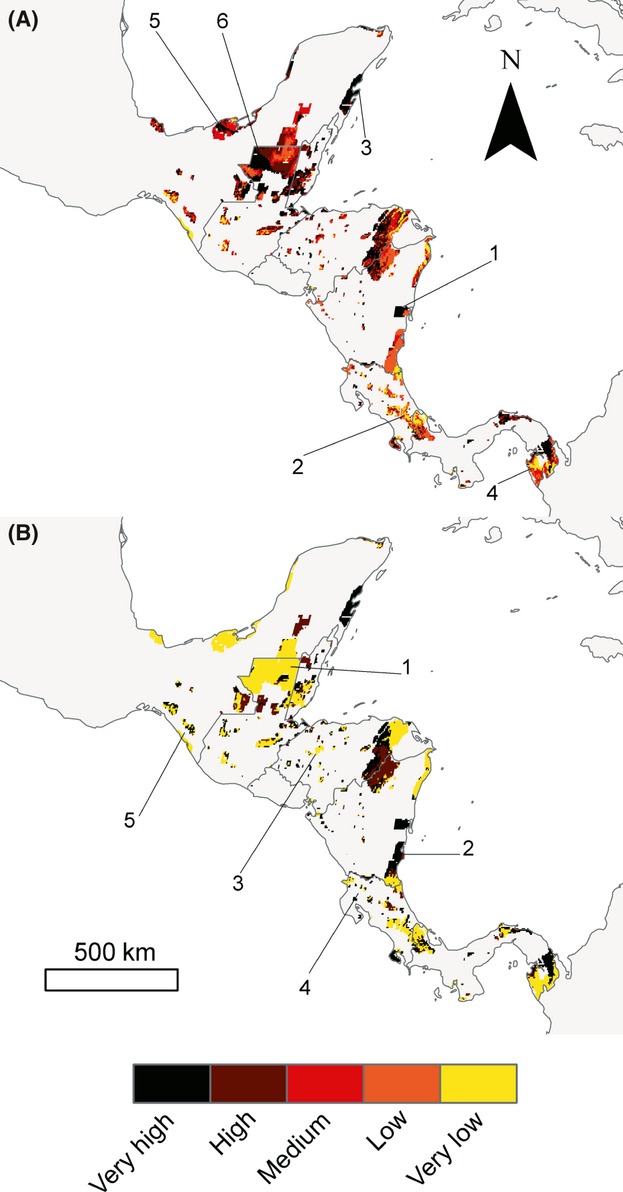
Impacts on climate change on protected areas without biological corridors (A) and difference of impacts with and without corridors (B) (Mollweide projection).

Impacts of climate change on PA are significantly associated with all variables tested (Table 2), except for the size of the area. The range of altitude and precipitation have the largest independent influence on the impact (24% and 25%, respectively, in Fig. 5A) followed by current temperature (14%), increase in temperature (14%), and decrease in precipitation (13%) and altitude (9%). Therefore, higher impacts are associated with flat (Fig. 6A-1 as opposed to Fig. 6A-2 on the mountains), hot, dry areas (e.g., the Yucatan Peninsula combines all factors for high impacts, Fig. 6A-3, as opposed to Fig. 6A-4). Also PA with bigger changes in future climate experience higher impacts (also, Fig. 6A-2), as do those in lowlands (Fig. 6A-5).

**Table 2:** Pearson correlations (coefficients = *C* and *P*-values = *P*) for variables associated with mean presence of PFT under climate change on protected areas (PA), benefit for protected areas from biological corridors (BC), and importance of biological corridors

	Presence on PA		PA benefit from BC		Importance of BC	
			
	*C*	IE (*Z*)	*C*	IE (*Z*)	*C*	IE (*Z*)
Area	0.05	0.07	−0.17	1.31	0.32	**12.34**
Altitude	0.21	**2.49**	0.17	**7.84**	−0.05	**1.96**
Altitude range	0.33	**7.57**	−0.17	**8.06**	0.20	**7.95**
Temperature	−0.22	**4.76**	−0.13	**5.82**	0.07	0.74
Increase in temperature	0.12	**4.74**	0.15	1.24	−0.12	**4.28**
Precipitation	0.27	**8.08**	−0.12	0.37	0.03	1.01
Decrease in precipitation	0.29	**4.78**	0.11	0.48	−0.12	**7.99**

*Z*-scores for the independent importance (IE) are given for variables used in the hierarchical partitioning protocol (those with *C* > 0.1); bold values denote variables with significant *Z*-scores above 1.65.

On average in the region, the possibility of having species suited to the 2050 climate in PA depends on policy scenarios (i.e., the presence of corridors) but only for PFT with high dispersal rates (types E to G). For plant types with low dispersal rates (types A to D), the corridors do not influence significantly their future presence in PA because climate changes across PA are faster than low dispersal PFTs can move.

### Contribution of corridors to impacts reduction

The contribution of corridors in reducing impacts on PA (i.e., the difference in impacts with or without corridors) depends on climate scenarios (higher contribution for faster climate change) on dispersal rates (higher contribution to faster rate PFTs), but not on the vegetation model used.

Some PA (i.e., the Mayan Biosphere Reserve, Fig. 1B) will be highly impacted by climate change (Fig. 6A-6), but corridors do not reduce these impacts (low difference in Fig. 6B-1). The benefit that corridors provide to a protected area depends on its altitude range (32% of independent effects, Table 2), its altitude (26%), and current temperature (21%); other variables were not significant (Fig. 5). PA that benefit the most from corridors have lower range in altitude (Fig. 6B-2 as opposed to Fig. 6B-3), located in highlands (Fig. 6B-4) and with lower temperatures (Fig. 6B-5).

**Figure 7:**
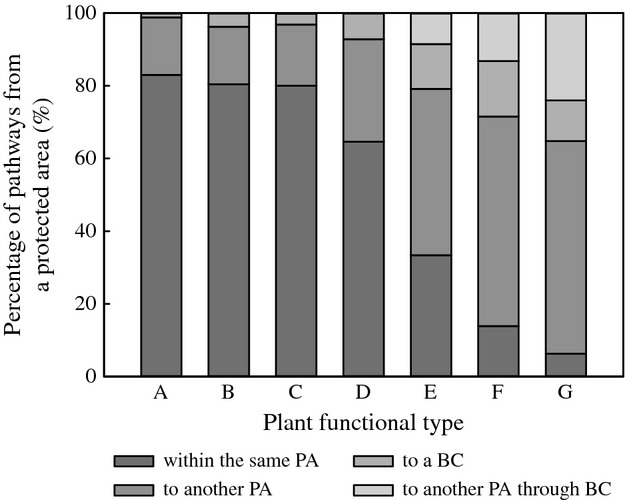
Distribution of the destination of dispersal paths originating from a protected area, for a range of dispersal rates from slow (A) to fast (F) functional types (PA, protected area; BC, biological corridor) (pathways outside PA or BC are not shown).

For slow moving PFTs (A to D), almost all migration pathways (93–99%) are within the same protected area and within a group of connected PA (Fig. 7). For fast-moving PFTs (G), up to 24% of the pathways end in another protected area through a corridor but a majority of the pathways (up to 58%) start in one protected area and end in another without leaving a protected area. For fast PFT (E to G types), between 21% and 35% of PFT in PA use a corridor at any time step for dispersal (either to move to another protected area or to end up in a corridor).

**Figure 8:**
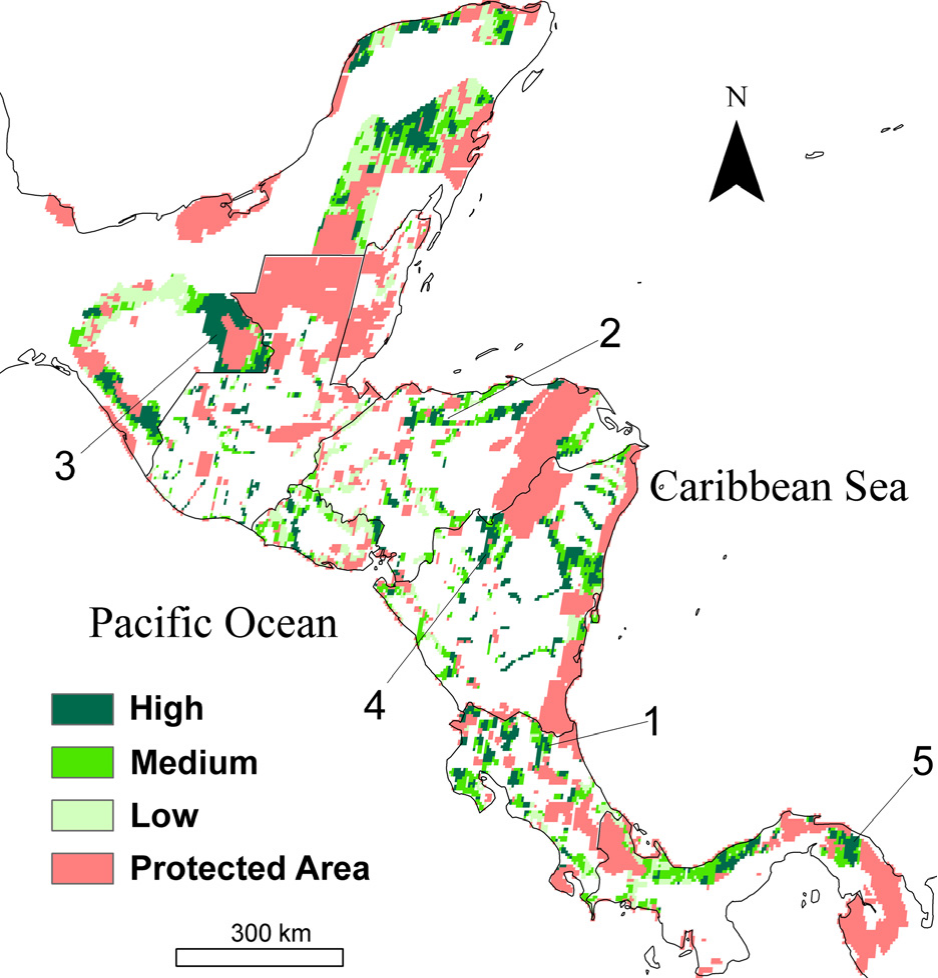
Biological corridors and their importance (low, medium, high) for species dispersal between protected areas (Mollweide projection).

Variables explaining corridors importance are area (31% of independent effects), altitude range (21%), decrease in precipitation (21%), and increase in temperature (12%) and altitude (7%) (Fig. 5). Therefore, most of the migration of (fast) plants between PA goes through corridors that are relatively large, with a larger altitudinal gradients and small changes in precipitation (Table 2). To a minor extent, also those with smaller increase in temperature and at lower altitude. For example, altitudinal corridors of high importance are found in northern Costa Rica (Fig. 8-1), with large areas in Honduras or Mexico (Fig. 8-3), with small changes in climate in Honduras (Fig. 8-2) and Nicaragua (Fig. 8-4) or at lower altitude (Fig. 8-5).

## Discussion

Patterns in velocity of climate change are similar to those found by Loarie et al. (2009) with high velocities in flat areas and slower over mountains where the increase in temperatures can be offset by short distance increases in altitude. Mapped velocities of climate change show areas where some PFTs, even under ideal dispersal conditions, will not be able to keep up with the velocity of climate change. We found that PFTs, except for the fastest, will face rates of change faster than their dispersal capacity. Furthermore, areas where the slowest PFTs could match the velocity of change are located on mountain tops and therefore dispersal will not be possible. Particularly for slower PFTs, comparing dispersal speed with the velocity of climate change might be misleading as the velocity estimates depend on the spatial resolution and kernel used (Loarie et al. 2009) that might not capture smaller scale gradients (where, e.g., an increase in temperature can be offset by a short-distance movement to higher lands). For the fastest PFTs the scenarios are more optimistic as they could potentially move on most of the region. This is also true for our modeling results because as species become slower impacts are higher. It is important to note that this comparison is based on annual means of precipitation and temperature, although other factors such as seasonal and interannual criteria to estimate the velocity of climate change could be of importance.

Migration involves several processes (e.g., fecundity, dispersal, recruitment, and population growth) and our approach is limited as it only focuses on dispersal to assess the impacts and role of BC to facilitate this process. Furthermore, accounting for the minimum range size for a species to be viable (Phillips et al. 2008), corridor configurations that link climate refugia sites, the colonizing capacity of species (Vos et al. 2008), or nonanalog climates (Araújo et al. 2011) are also necessary. Further steps also require developing recommendations for field implementation, for example, Hole et al. (2011) defines site management goals depending on the number of species persisting or coming in and out (of a site) to define alternatives for adaptation strategies. Finally, conservation planning should not disregard other threats (such as habitat loss), but work on integrated approaches (Hannah 2011).

Assessment of impacts on PA depends on the climate model selected; therefore, further similar studies should assess a larger number of climate scenarios. Furthermore, the metric used to measure and compare impacts (i.e., with and without corridors as in Fig. 6) shows a relative gradient whose ecological meaning remains unknown and therefore highly limits its interpretation beyond its relative ranking. For example, it is not possible to know the difference between high and very high impacts or whether how important a low impact is in terms of the ecological functions of ecosystems in a protected area.

PA with no altitudinal gradients are the most impacted when located at the lower end of the altitude and climate (temperature and precipitation) ranges, as under future drying trends, these areas will have no source of species coming from drier/hotter areas and no gradients to develop suitable climates for their native species in future scenarios. As expected, lower impacts are found under smaller changes in climate.

PA that do not have a relative large elevation range or located in cool highlands benefit from corridors that provide the gradient needed for dispersal of species (from hotter and lower altitudes) to their new suitable climates (on the benefited areas). Accordingly, PA already having an elevation gradient receive a relatively smaller benefit from corridors as they can already provide for future suitable climates within their boundaries.

Corridors in this experiment contribute to migration by extending the area of contiguous vegetation, through a sharp increase (strong conservation policy) or decrease (weak conservation policy) in pixels suitable for dispersal (i.e., covered by natural vegetation). Pearson and Dawson (2005) also found that the amount of suitable habitat is particularly important for successful migration by LDD and that landscape fragmentation becomes important as dispersal is slower. Therefore, corridors are important for slower species (if the velocity of climate change is not too high) due to reduced landscape fragmentation, while for LDD they play a role in increasing suitable habitat (as do PA) along the climate gradient. Slower species survival will depend on their capacity to persist in different climates (adaptation was not treated explicitly in our study) or on assisted migration efforts (Pearson and Dawson 2005), although existing research gaps on the associated risks for the later limit the definition of policies (McLachlan et al. 2007).

Results from Engler et al. (2009) are in contradiction with ours, they found small differences on species extinctions between slow and fast dispersal rates. This is probably due to the larger size of our study area and a terrain that combines mountain and flat areas. In our case, also the range of accumulated dispersal distance is larger (100 km in a 10-year time step for the fastest PFT under LDD), but within ecological timescales proposed for several LDD mechanisms that operate over nonforested areas (Nathan et al. 2008); therefore, LDD can become an important species trait. Additionally, our results do not depend on the changes in suitable areas for each PFT as all pixels contain the same dispersal rates. However, we assumed that all species are vegetation type–specific and we did not account for the possibility of vegetation types sharing species (Malcolm et al. 2006) which could overestimate impacts.

Physical templates are gradients (i.e., topographic) stable in time compared to management timelines that have been proposed for robust strategies of conservation under highly uncertain futures (Hagerman et al. 2010). Our explanatory variables for important corridors (those with large areas, large altitudinal range, and smaller change in climate in lowlands) could serve as a first step to define physical templates for the design and selection of priority corridors as means to conservation goals under climate change. Precautions should be taken as these findings are not general rules about corridors and only apply to the context of the Mesoamerican region for the climate ranges and policy scenarios analyzed.

Drier PA are those most impacted by climate change and they will be also needed as a source of species as ecosystems are projected to shift to drier conditions in Mesoamerica (Imbach et al. 2012). In some cases this will require cross-country coordination and monitoring (Hannah et al. 2002), for example, PA in coastal northern Yucatan (currently covered by dry and very dry forests) could help by providing a reserve of species adapted to future drier conditions in northern Guatemala. PA in northern Guatemala will be highly impacted while current corridors have a low importance. Furthermore, drier ecosystems imply a higher risk of fires (Lewis 2006) that could further threaten biodiversity. Finally, Mesoamerica is a hotspot for novel climates (Williams et al. 2007) where ecosystems with new species assemblages will develop based on species persisting in situ and dispersal from other areas.

Accounting for future threats to define priority areas can increase the effectiveness of conservation efforts (Spring et al. 2010), therefore further studies could try to account for important corridors for species dispersal when defining conservation priorities in Mesoamerica. Our results also show that species will spend a considerable amount of time in or passing through corridors and therefore its role in providing protection for species should be accounted for.

The work emphasized on the role of corridors by considering plant migration as a useful process for plant adaptation but migration of invasive species could be a threat to biodiversity. Non-native species could benefit from altered climatic constraints and become invasive, changes in the distribution range of suitable areas for invasive species can facilitate their dispersal to new areas and competitive interaction with new species assemblages can reduce the abundance of native species (Hellmann et al. 2008).

Further work should include movement through other land uses in the landscape (i.e., complex and diverse agroforestry systems) given their importance for noncultivated plant species (Bhagwat et al. 2008) and as a productive alternative for corridor areas. Dyer (1995), for example, defined a gradient of probabilities for successful colonization across landscape classes and their degree of disturbance. In our study, some productive systems are identified as forests (e.g., coffee agroforestry systems under forests in Guatemala) as they are difficult to distinguish from forests, but others are not. Our assumption that dispersal occurs only over forest areas leads to an overestimation of impacts, particularly under diffusion process, as at least for some species, disturbed vegetation cover can also provide grounds for dispersal.

The scenarios developed could be used as a tool to develop a shared understanding of the implications of climate change in order to plan for adaptation of the Mesoamerican socioecological system (Brooke 2008).

## Conclusions

Our results show the vulnerability of PA in the MBC facing climate change based on broad assumptions on the dispersal capacity of species. We found that dry lowland PA are most vulnerable and that under future velocities of climate change, faster species will benefit from corridors for dispersal. Of particular importance are large altitudinal corridors. Our approach can be useful to identify vulnerable PA and prioritizing conservation planning in a context of climate change in high species richness areas.
